# Visual perspective taking is not automatic in a simplified Dot task: Evidence from newly sighted children, primary school children and adults

**DOI:** 10.1016/j.neuropsychologia.2022.108256

**Published:** 2022-05-07

**Authors:** Paula Rubio-Fernandez, Madeleine Long, Vishakha Shukla, Vrinda Bhatia, Pawan Sinha

**Affiliations:** a University of Oslo, Norway; b Massachusetts Institute of Technology, USA

**Keywords:** Visual perspective taking, Theory of Mind, Automatic processes, Interference, Executive control

## Abstract

In the Dot task, children and adults involuntarily compute an avatar’s visual perspective, which has been interpreted by some as automatic Theory of Mind. This interpretation has been challenged by other researchers arguing that the task reveals automatic attentional orienting. Here we tested a new interpretation of previous findings: the seemingly automatic processes revealed by the Dot task result from the high Executive Control demands of this verification paradigm, which taxes short-term memory and imposes perspective-switching costs. We tested this hypothesis in three experiments conducted in India with newly sighted children (Experiment 1; N = 5; all girls), neurotypical children (Experiment 2; ages 5–10; N = 90; 38 girls) and adults (Experiment 3; N = 30; 18 women) in a highly simplified version of the Dot task. No evidence of automatic perspective-taking was observed, although all groups revealed perspective-taking costs. A newly sighted child and the youngest children in our sample also showed an egocentric bias, which disappeared by age 10, confirming that visual perspective taking develops during the school years. We conclude that the standard Dot task imposes such methodological demands on both children and adults that the alleged evidence of automatic processes (either mindreading or domain general) may simply reveal limitations in Executive Control.

## 1. Introduction

**Fig. 1.**
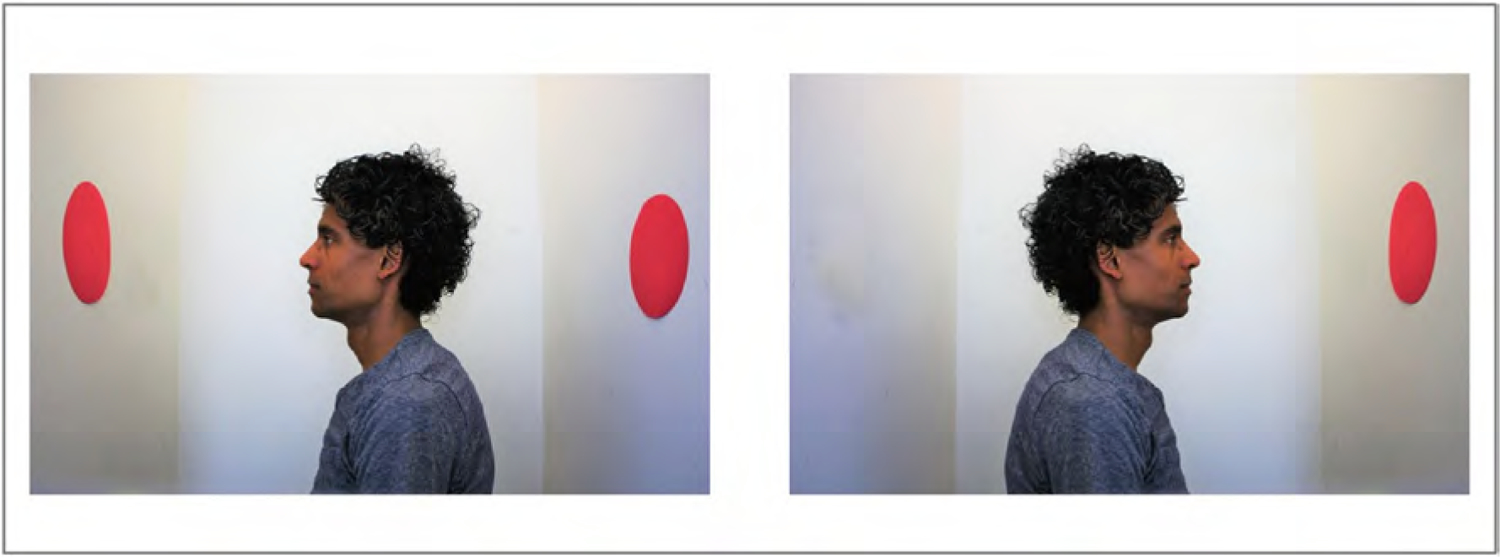
Sample pictures from the simplified Dot task that was administered longitudinally to newly sighted children (ages 9–18; N = 5) and cross-sectionally to neurotypical children (ages 5–10; N = 90) and adults (ages 21–30; N = 30). The picture on the left is from an incongruent trial (i.e. the number of dots is different from the man’s vs. the participant’s perspective), whereas the picture on the right is from a congruent trial (i.e. both the man and the participant see the same number of dots).

Visual perspective taking (VPT) is a cornerstone of Theory of Mind development (Wellman, 2014). In Piaget and Inhelder’s (1956) landmark study, 4-year-olds failed to adopt the visual perspective of a doll who was facing a model of three mountains from a different angle, selecting the view that matched their own, egocentric perspective. After decades of intense research, the most recent visual perspective-taking task was designed by Samson et al. (2010), who presented adults with an avatar in profile, which was facing one of two opposite walls. Participants had to indicate how many dots were on the walls, either from their own perspective or from the avatar’s. In congruent trials, the participant and the avatar saw the same number of dots (i.e. all dots were on the wall that the avatar was facing), whereas in incongruent trials, the participant could see more dots than the avatar (i.e. some of the dots were on the wall behind the avatar). For an illustration from the present study, see Fig. 1.

Like the 4-year-olds in Piaget and Inhelder’s study, the adults in Samson et al. (2010) also revealed an *egocentric bias*, showing lower accuracy rates and slower responses in incongruent trials than in congruent trials when asked to adopt the avatar’s visual perspective. That is, their own view of the scene interfered with their calculation of the avatar’s perspective. More interesting for the Theory of Mind community, these participants also revealed an *altercentric bias*: when asked to perform the task from their own visual perspective, participants also showed poorer accuracy and slower response times (RTs) when the avatar had a different perspective from their own. That is, even when the avatar’s view was immaterial to their task, participants seemed to automatically calculate the avatar’s perspective.

Analogous to the much debated false-belief task (Apperly and Butterfill, 2009; Wellman, 2014), Theory of Mind researchers have proposed both rich and lean interpretations of the altercentric interference observed with the Dot task. Samson et al. (2010) interpreted the original findings in line with Apperly and Butterfill’s (2009) two-system account of Theory of Mind: humans can quickly and efficiently determine what another person sees, guided by direct cues such as their line of gaze. However, more complex forms of VPT (e.g., determining how a mountain range looks from some else’s perspective; Piaget and Inhelder, 1956) require more Executive Control and do not appear to be automatic (see Flavell et al., 1981). Samson and colleagues “avoided referring to these simple perspective-taking processes as “automatic” because automaticity is difficult to establish without a more exhaustive investigation of the range of circumstances in which such phenomena are observed” (2010:1264).

More recently, a number of studies replicated and extended the results of Samson et al. (2010), which led to the conclusion that VPT is indeed an automatic process in humans (see Qureshi et al., 2010; Nielsen et al., 2015; Ferguson et al., 2017, 2018; Furlanetto et al., 2016; Simpson and Todd, 2017). Under this rich interpretation, the altercentric interference observed in these studies has been taken as evidence that the mere presence of an avatar automatically triggers VPT (Surtees and Apperly, 2012; 2016).

However, the interpretation of the above findings as *automatic mindreading* has not gone unchallenged. Santiesteban et al. (2014) observed similar interference when replacing the avatar with a non-social directional cue, such as an arrow. In addition, Cole et al. (2016) and Conway et al. (2017) observed altercentric interference when the avatar’s view had been blocked, challenging the conclusion that participants were calculating the avatar’s visual perspective (for a commentary, see Catmur et al., 2016). The results of these studies have been interpreted as evidence that the altercentric interference observed with the Dot task results from domain-general processes, such as *attentional orienting*.

The present study tested the rich interpretation of the altercentric interference observed with the Dot task as evidence of automatic VPT in humans (Qureshi et al., 2010; Nielsen et al., 2015; Ferguson et al., 2017, 2018; Furlanetto et al., 2016; Simpson and Todd, 2017). To this end, we recruited a group of participants with a unique visual profile: children with late sight onset, who have been treated from congenital cataracts in recent years. In adapting the Dot task for this population, we also put forward and tested an alternative interpretation of earlier findings: the altercentric interference observed with the Dot task may be a product of the high Executive Control demands of the task, rather than a measure of automatic processes (either VPT or attentional orienting). This third interpretation is therefore different from the two alternative accounts that have dominated this debate to date (Theory of Mind vs domain-general processes), and is in line with recent work investigating what exactly Theory of Mind tasks measure (Quesque and Rossetti, 2020).

### 1.1. Developmental evidence of automatic visual perspective taking

Surtees and Apperly (2012) adapted the Dot task to investigate VPT in children aged 6–10 years and adults. They argued that developmental work was important to determine whether VPT changes as children gain social experience and cognitive resources. Surtees and colleagues predicted that, if the results of Samson et al. (2010) revealed ‘original automaticity’, children should also reveal altercentric interference when performing the Dot task from their own perspective. Alternatively, if the automatization of VPT requires social experience, children should differ from adults in their perspective-taking skills.

Surtees et al. observed evidence of both egocentric and altercentric interference at all ages. Interestingly, egocentrism did not decline during development, suggesting that improvements in visual perspective-taking in adulthood are not brought about by a reduction in one’s egocentric bias. As Surtees et al. argue, “egocentrism in adults does not merely resemble egocentric phenomena observed in children but, for simple perspective taking at least, reflects the same underlying cognitive processes: We cannot help having our own perspective and this interferes with judgments about the perspective of others” (2012:458).

In addition, altercentric interference was only observed in their first experiment with an avatar: when Surtees and colleagues changed the avatar for a non-social directional cue in their second experiment, participants performed comparably in congruent and incongruent trials. This difference led the authors to conclude that VPT is automatically triggered by the presence of an agent – and not by non-social directional stimuli.

Surtees and Apperly (2012) used a mixed-trial design, in which children had to change perspectives across trials. Before the avatar and the dot(s) appeared on the screen, children were told how many dots they themselves or the avatar could see (depending on the condition), and they had to respond Yes or No according to what they saw in the next slide. A critical challenge to the conclusions of Surtees and Apperly (2012) is that the children in their study may have had problems ignoring the avatar’s perspective in those trials when they had to perform the task from their own perspective because in other trials, the avatar’s perspective was critical. In other words, children may have experienced *perspective-switching costs* that would not have affected their performance in a different task design.

Surtees and Apperly (2012) ruled out the possibility that their results could be due to a carryover effect across different types of trials because they did not observe the same type of interference when they employed a non-social directional cue in their second experiment. Since both experiments used a mixed-trial design, carryover effects should have been observed in both versions of the task (social and non-social). However, more recent studies with adults have shown that using a block design (where participants do not change perspectives from one trial to the next) reduces the degree of altercentric interference observed with the Dot task (Ferguson et al., 2017; del Sette et al., 2021).

Ferguson et al. (2017) and del Sette et al. (2021) observed that altercentric interference could be reduced, or even eliminated when adult participants performed the task from their own perspective in consecutive trials. These results seem to contradict the automaticity claims made in other studies using the Dot task (e.g., Surtees and Apperly, 2012; 2016; Furlanetto et al., 2016). However, Ferguson et al. (2017) interpreted their results as consistent with the rich interpretation of previous studies because their eye-tracking data revealed different fixation patterns when participants were performing the task from their own perspective and from the avatar’s.

The question of whether the egocentric and altercentric biases observed with the Dot task result from the Executive Control demands of the task (rather than from automatic Theory of Mind processes) is relevant to a recent review of Theory of Mind tasks by Quesque and Rossetti (2020). These authors argue that most classic tests, including the Dot task, do not require participants to represent another’s mental states. Instead, standard tasks measure lower-level processes that do not directly test for Theory of Mind. Quesque and Rossetti (2020) conclude that more attention should be paid to the methods used in Theory of Mind research in order to improve our understanding of the underlying concepts.

### 1.2. Methodological considerations and aims of the present study

The first aim of our study was to use a highly simplified version of the Dot task with newly-sighted children, as this populations has previously provided a way to address questions of nativism vs empiricism (Held et al., 2011; Sinha et al., 2020). As part of Project Prakash (Sinha and Held, 2012; Sinha, 2013), we tested five girls in India ranging in age from 9 to 18 years (mean: 12.4 years), who had been treated for blindness due to dense bilateral congenital cataracts (for details of the factors used to determine congenitality, see Ganesh et al., 2014). Each child underwent cataract removal surgery in recent years and received intraocular lens implants. These children are currently receiving tailored education at a specialized boarding school in Delhi.

The debate around the automaticity of VPT has often considered two theoretical possibilities: adults may come to automatically adopt other people’s visual perspective through extensive social interaction; alternatively, basic VPT may be an innate Theory of Mind capacity that can already be observed in infancy (Qureshi et al., 2010; Surtees and Apperly, 2012; 2016; Furlanetto et al., 2016). The Prakash children investigated in this study offer a test-case of these hypotheses, having gained vision later in childhood. If these children reveal altercentric interference in a simplified version of the Dot task, their performance would suggest original automaticity (i.e. an automatic response that is present from birth; Surtees and Apperly, 2012), since their experience with VPT was limited during early development.

It must be noted, however, that while the visual acuity of the Prakash children normally improves significantly after surgery, their vision is often compromised. Therefore, low visual acuity could also affect Prakash children’s performance in the Dot task, above and beyond their Theory of Mind abilities. Bearing these potential limitations in mind, the present investigation was conceived as an exploratory study, looking at the development of VPT in Prakash children and neurotypical school children.

The second aim of the study was methodological: in order to make the Dot task suitable for Prakash children, we had to greatly simplify the original design. This methodological approach was both practical and experimental in nature: a highly simplified design allowed us not only to run the task with newly-sighted children, but also investigate whether the perspective interference observed in previous studies was an artifact of the high Executive Control demands of the original design. Such a finding would cast doubts on the validity of the Dot task as a measure of involuntary processes – whether mentalizing or domain general.

To simplify the Dot task, we made two key changes to the original design. First, we used a block design in which participants were first asked to perform the task from their own perspective, and only in the second half were they asked to perform the task from the agent’s perspective. The results of Ferguson et al. (2017) and del Sette et al. (2021) with adults suggest that this block design may eliminate the altercentric interference observed in other studies. The rationale for administering the self-perspective version of the task in the first block of trials was to avoid making the agent’s perspective artificially salient prior to testing allocentric interference.

A consistent finding in all studies using the Dot task is that allocentric interference (i.e. the difficulty of ignoring another agent’s visual perspective when performing the task from one’s own) is weaker than egocentric interference (i.e. the difficulty of ignoring one’s own visual perspective when adopting another agent’s). In fact, the debate around the Dot task has focused on the nature and the degree of allocentric interference observed with this paradigm, rather than on the egocentric interference it also elicits (for a recent review, see del Sette et al., 2021). We therefore prioritized the accuracy of our measure of allocentric interference by administering the self-perspective version of the task in the first block of trials.

Regarding egocentric interference, we wanted to see if a simplified design would reveal a reduction of this egocentric bias across ages – contrary to the results of Surtees and Apperly (2012) using the original, more demanding task design. Since both child and adult participants in our study performed the perspective-taking task in the second block of trials, any developmental patterns observed across the two age groups should be reliable.

The second change that we made to the original Dot task was to avoid loading participants’ working memory by giving them a number of dots and a perspective ahead of each display (e.g., ‘You see three’ or ‘He sees three’) and asking them to verify that information in view of the ensuing display. It must be noted that this feature of the original task design poses high demands on short-term memory, while making the Dot task a *verification paradigm* – rather than a simple perspective-taking task, as it is normally described. To avoid such methodological demands, we asked participants to directly indicate whether there were one or two dots in the scene (Block 1), and whether the man in the picture could see one or two dots in the same scene (Block 2). This is an important modification that greatly reduces the working memory demands of the Dot task, while more directly tapping into VPT.

In Experiment 1, we administered the simplified Dot task to five Prakash girls in four testing sessions, to see if Prakash children would develop strategies to improve their VPT over time. The same simplified task was administered to neurotypical school children (ages 5–10) in Experiment 2 and to adults in Experiment 3. Sample size in the first experiment was determined by the number of Prakash girls attending the boarding school at the time of testing (N = 5), while in the second and third experiments, sample size was established a priori according to previous studies (N = 30 per age group). All experiments were conducted in India. All measures, manipulations, and exclusions from the study are disclosed in the experimental report.

## 2. Experiment 1

### 2.1. Methods

#### 2.1.1. Participants

Five Prakash girls (ages: 9, 10, 12, 13, 18; M = 12.4) were recruited from a boarding school in Delhi (India). They were tested four times, at 4-day intervals during a period of 2 weeks. The study was approved by MIT’s IRB under protocol ‘Development of Visual Perception’ (\#:0403000050R016). See Table 1 for demographic and clinical information.

The clinical information in Table 1 reveals that four of the Prakash girls in our sample took part in our study 1–2 years after surgery. However, Participant D was tested on the Dot task 7 years after treatment, which is a considerably longer delay. While we readily acknowledge that the time since surgery affects these participants’ experience with VPT (and could therefore potentially affect their performance in the Dot task), all the girls in our sample suffered from congenital cataracts, which has been shown to affect visual cognition even after receiving early treatment (Maurer et al., 2007; Maurer, 2017). Prakash children therefore offer an interesting test case for the nature of VPT (as innate or emerging), even if their individual profiles may not generalize across the whole group.

Regarding the measures reported in Table 1, it is also important to bear in mind that visual acuity, while indicative of visual gains after treatment, is not the only measure of functional vision. As Ben-Ami et al. (2022) have recently put it, visual acuity may be used as “a measure of low-level visual capacity in the absence of alternative measures, but should be considered as an impoverished metric that is not sufficient as sole estimator of post-surgical changes in functional vision.” In our sample, Participant C did not show an improvement in visual acuity 6 months after surgery. However, when tested without visual correction (namely glasses, which tend to improve visual acuity in this population), Participant C showed a slight improvement in visual acuity 1 month post-test (2.58 > 2.35 logMAR).

#### 2.1.2. Materials, task design and procedure

A series of 6 pictures were taken, showing a man in profile standing between two close walls (see Fig. 1 for sample pictures). One or two large red dots were shown on the walls, aligned with the man’s head. To facilitate visual perception of the scene, the space depicted in the pictures was considerably narrower than the 3D spaces that were computer-generated in avatar versions of this task. Thus, only the head and shoulders of the man were shown in the pictures (rather than a full body silhouette seen from a distance). The pictures fully counterbalanced the wall that the man was facing (right-left), with two pictures showing one dot in front of the man, two pictures showing two dots in front of the man, and two pictures showing one dot in front and one dot behind the man. In this simplified design, when dots were shown on both walls, the agent’s and the participant’s perspectives were incongruent; otherwise, they were congruent.

The task was built using jsPsych (De Leeuw, 2015), a JavaScript library. Prior to the start of the task, a series of 20 practice trials were delivered in a random order showing only one or two dots on the walls (i.e. without the man standing in between the walls). Children were asked to press the P and Q keys on their keyboard to indicate one or two dots, respectively. The actual task employed a two-block design, with each block including 40 trials and a pause built in between the two blocks. In the first half of the task, children were asked to indicate how many dots they saw in the picture, as fast and accurately as they could. Then, during the break, they were told that they were going to change perspective: now they had to indicate how many dots the man could see from where he was standing.

Children used their right hand to indicate one dot (letter P on their keyboard) and their left hand to indicate two dots (letter Q on their keyboard). In Block 1, trials showing one dot corresponded with the Congruent condition (i.e. the dot appeared in front of the man), while trials showing two dots corresponded with the Incongruent condition (i. e. one of the dots appeared behind the man). A possible right-hand bias would therefore augment any allocentric interference observed in Block 1 (i.e. slower responses in the Incongruent condition). In Block 2, the correct response in the Congruent condition was 2 (i.e. two dots appeared in front of the man), while the Incongruent condition required responding 1 (i.e. one dot was in front of the man, while a second dot appeared behind). A possible right-hand bias would therefore reduce any egocentric interference observed in Block 2 (i.e. faster responses in the Incongruent condition). The division of keys and responses for each condition therefore favored ‘Theory of Mind performance’, potentially increasing allocentric interference and reducing egocentric interference in each block. Failing to see such patterns can therefore not be due to a potential right-hand bias.

The task was hosted on Cognition (https://www.cognition.run/) and administered remotely with the help of an assistant at the boarding school where the Prakash girls are studying. The experimenter contacted the assistant and the participant via Zoom. They were provided a link to the task and were asked to share their screen. Before the practice session started, the assistant ensured that the participant placed their fingers on the correct keys. Audio feedback was given for each practice trial (ringing applause or slightly downbeat sound). Once they completed the 20 practice trials, participants were told that they still had to indicate how many dots were on the screen, but now a man would be standing in between the two walls. In the second block of trials, children were asked to indicate how many dots the man could see using the same two keys. No feedback was given during the two trial blocks.

## 3. Results

Run 3/Block 1 data from Participant E had to be discarded because she was pressing the wrong keys. With the exception of those data, accuracy rates were almost at ceiling in the four runs of the task (Mean percentage of correct responses across participants: 97% (Run 1), 97% (Run 2), 95% (Run 3) and 97% (Run 4)). We interpret the high accuracy rates as evidence that the visual materials and the task were suitable for the Prakash girls in the study.

Given the individual variability in visual acuity and task performance, each child was treated as a case study, with separate statistical analyses performed on each child’s data. Using linear mixed effects regression, we modelled the outcome variable of RTs (for accurate responses) with Condition (Congruent, Incongruent), Block (Block 1 = Self perspective, Block 2 = Agent’s perspective), Run (Run 1, Run 2, Run 3, Run 4) and their interactions as predictor variables. Run was entered as a scaled continuous predictor and deviation coding was used for Condition (Congruent = −0.5, Incongruent = 0.5) and Block (Block 1 = −0.5, Block 2 = 0.5). The model was fit with the maximal random effect structure for participants and items (Barr et al., 2013).

For two of the participants, model results did not reveal any significant main effects or interactions (for the full model outputs, see Supplementary Materials). Model results for the other three participants are reported below.

### 3.1. Participant A

There was a significant main effect of Run for Participant A (p = .0025), who exhibited faster RTs as the runs progressed (for the full model output, see Table 2).

### 3.2. Participant C

**Fig. 2.**
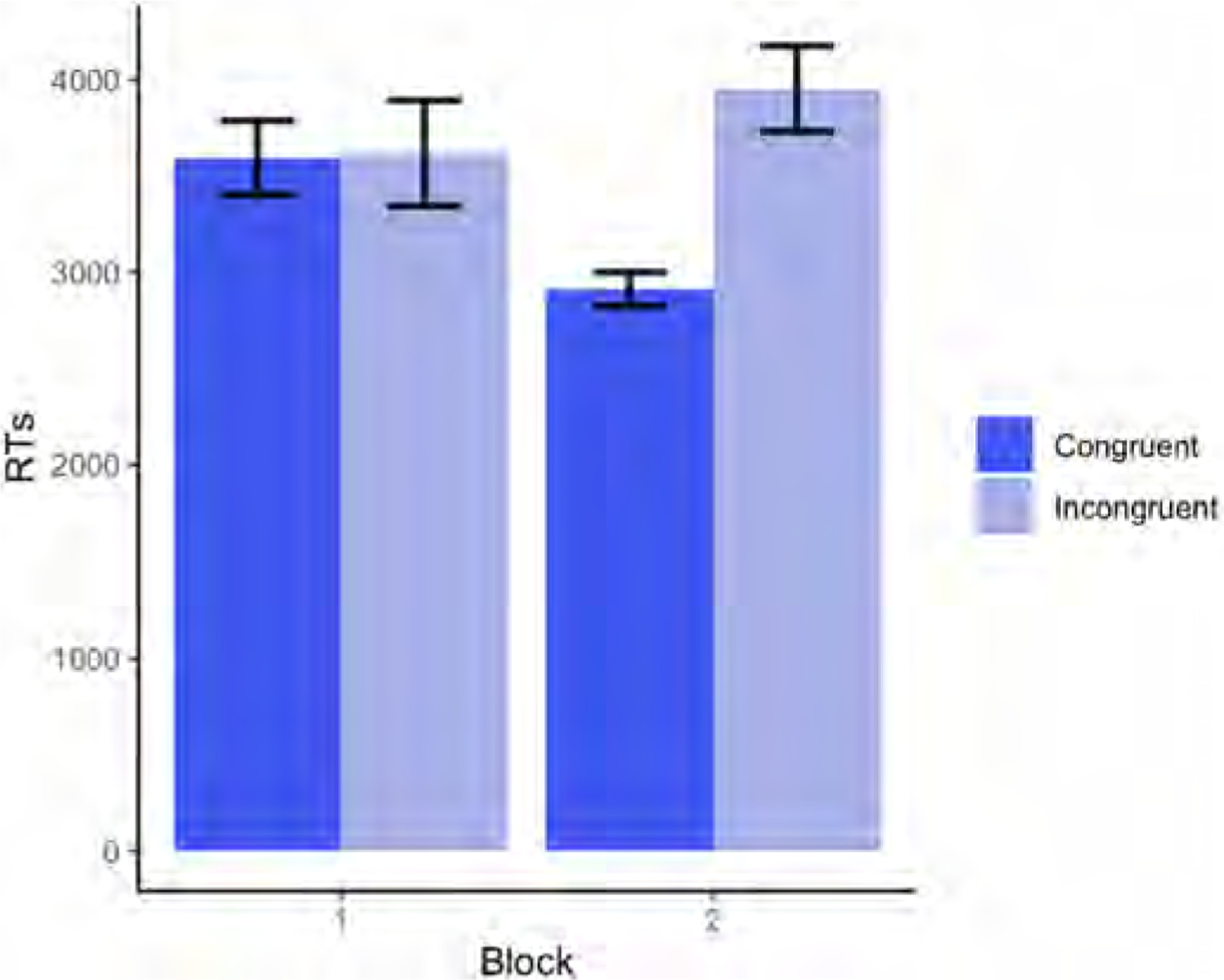
Average RTs (in milliseconds) on congruent and incongruent trials split by Block (Block 1 = self-perspective task; Block 2 = perspective-taking task) for Participant C in Experiment 1.

Results revealed a significant main effect of Run for Participant C (p = .0210), who exhibited faster RTs as the runs progressed (for the full model output, see Table 3). There was also a significant main effect of Condition (p = .0155), with faster RTs in congruent than incongruent trials. Finally, there was a significant Block × Condition interaction (p = .0196), driven by a significant difference between congruent and incongruent trials in Block 2 (p < .001), but not in Block 1 (p = .962) (see Fig. 2).

### 3.3. Participant E

Results revealed a significant main effect of Run for Participant E (p = .0159), who exhibited faster RTs as the runs progressed (for the full model output, see Table 4). There was also a marginal Block × Run interaction (p = .0644), with the difference between blocks decreasing (numerically) as runs progressed.

## 4. Discussion

The high accuracy rates observed in the five Prakash girls in Experiment 1, and the significant effect of Run observed in three of the girls (with RTs reliably decreasing across runs) suggest that the visual materials and task design were suitable for newly sighted children. However, the RT analyses only revealed a significant interaction for Participant C, whose performance was comparable in congruent and incongruent trials in Block 1, revealing no altercentric interference when performing the task from her own perspective. By contrast, Participant C’s RTs were significantly slower in incongruent trials than in congruent trials in Block 2, revealing egocentric interference when having to adopt the agent’s visual perspective.

It must be noted that Participant C’s performance revealed egocentric interference when taking the man’s visual perspective, rather than a general cost of changing task instructions in the second half of the task. That is, Participant C did not simply reveal slower RTs in the second half of the task (which could have suggested a general cost of changing to a perspective-taking task) but specifically slower RTs in incongruent trials relative to congruent trials in the perspective-taking condition.

While the above analyses were conceived as exploratory case studies, Participant C’s performance does not support the view that VPT is an innate human ability that operates automatically from infancy (Surtees and Apperly, 2012; 2016). If that were the case, we would expect to have seen altercentric interference when Partcipant C performed the task from her own perspective in Block 1. Alternatively, VPT may be an innate human ability but there might be a critical period when infants must be able to visually perceive their social interactions in order to trigger automatic VPT. For example, prior research with patients with late visual onset has shown that early experience with faces is crucial for the development of face processing (Maurer et al., 2007; Maurer, 2017). Therefore, a related critical period may affect VPT in Prakash children.

Experiment 2 aimed to investigate that possibility by testing neurotypical children between the ages of 5 and 10 years on the same simplified version of the Dot task. If VPT is an innate human ability that does not depend on social interaction beyond an early critical period, neurotypical children should show altercentric interference in the simplified Dot task.

## 5. Experiment 2

### 5.1. Methods

#### 5.1.1. Participants

Ninety children were recruited from a primary school in Delhi (India). The school serves middle-class families and teaches Kindergarten to Grade 12 (ages 4–17). Arrangements were made with the School Principal so that the experimenters would contact the teachers in the participating grades, who would in turn contact the parents of the children and set up a 3-way Zoom call. Thirty children were recruited from Grade 1 (5–6 years), thirty from Grade 3 (7–8 years) and thirty from Grade 5 (age 9–10 years).

#### 5.1.2. Materials, task design and procedure

The same materials, task design and procedure used in Experiment 1 were used again in Experiment 2. The task was conducted via Zoom, on a three-way call connecting a schoolteacher, the participating child (with a parent) and the experimenter.

## 6. Results

### 6.1. Accuracy analysis

Using logistic mixed effects regression, we modelled the binary outcome variable of Response Accuracy (Correct = 1, Incorrect = 0) with Condition (Congruent, Incongruent), Block (Block 1 = Self perspective, Block 2 = Agent’s perspective), Age, and their interactions as predictor variables. Age was entered as a scaled continuous predictor and deviation coding was used for Condition (Congruent = −0.5, Incongruent = 0.5) and Block (Block 1 = −0.5, Block 2 = 0.5). The model was fit with the maximal random effect structure for participants and items (Barr et al., 2013).

**Fig. 3.**
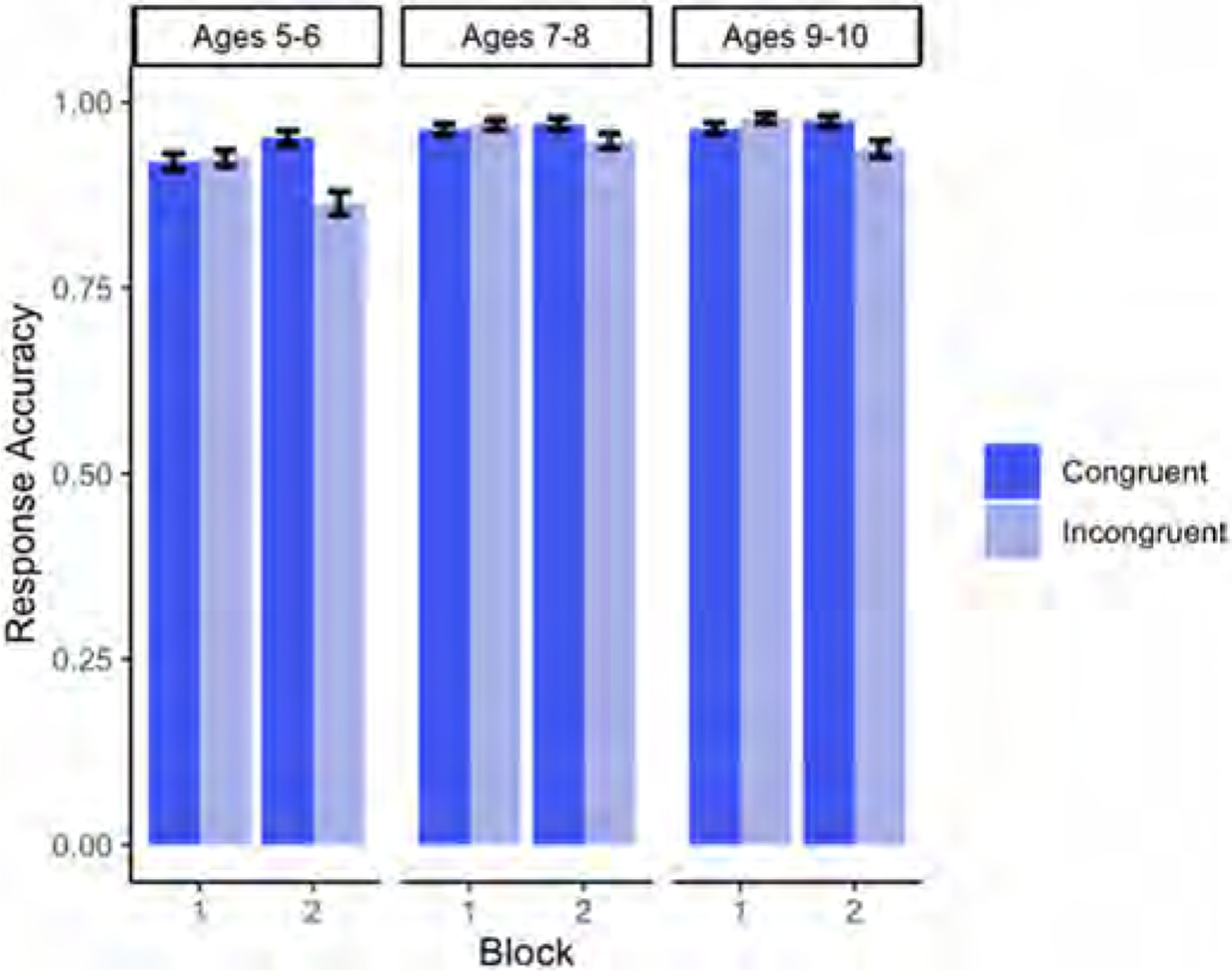
Mean proportion of accurate responses on congruent and incongruent trials from Experiment 2, split by Block (Block 1 = self-perspective task; Block 2 = perspective-taking task) and Age.

Descriptive statistics for each age group’s accuracy on congruent and incongruent trials split by block are shown in Fig. 3.

**Fig. 4.**
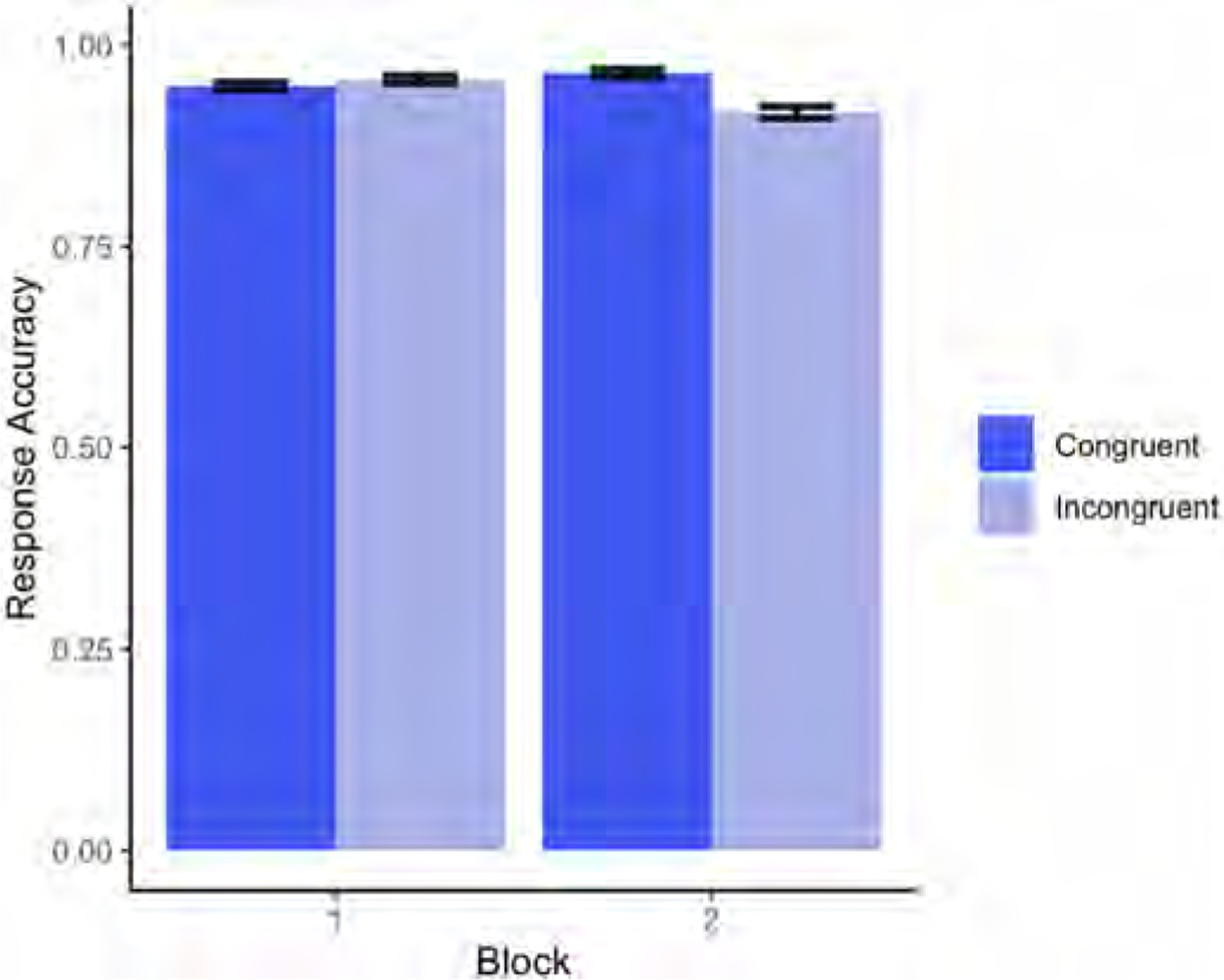
Block × Condition interaction for accurate responses in Experiment 2 (Block 1 = self-perspective task; Block 2 = perspective-taking task).

Our results revealed a significant main effect of Age (p < .001), in which accuracy improved as age increased (for the full model output, see Table 5). There was also a marginal main effect of Condition (p = .0501), with participants being (numerically) more accurate on congruent than incongruent trials. Finally, there was a significant interaction of Block x Condition (p < .001), driven by a significant difference in congruent and incongruent trials in Block 2 (p < .001), but not in Block 1 (p = .327) (see Fig. 4).

### 6.2. RT analysis

Using linear mixed effects regression, we modelled the outcome variable of RTs (for accurate responses) with Condition (Congruent, Incongruent), Block (Block 1, Block 2), Age, and their interactions as predictor variables. Age was entered as a scaled continuous predictor and deviation coding was used for Condition (Congruent = −0.5, Incongruent = 0.5) and Block (Block 1 = −0.5, Block 2 = 0.5). The model was fit with the maximal random effect structure for participants and items (Barr et al., 2013).

**Fig. 5.**
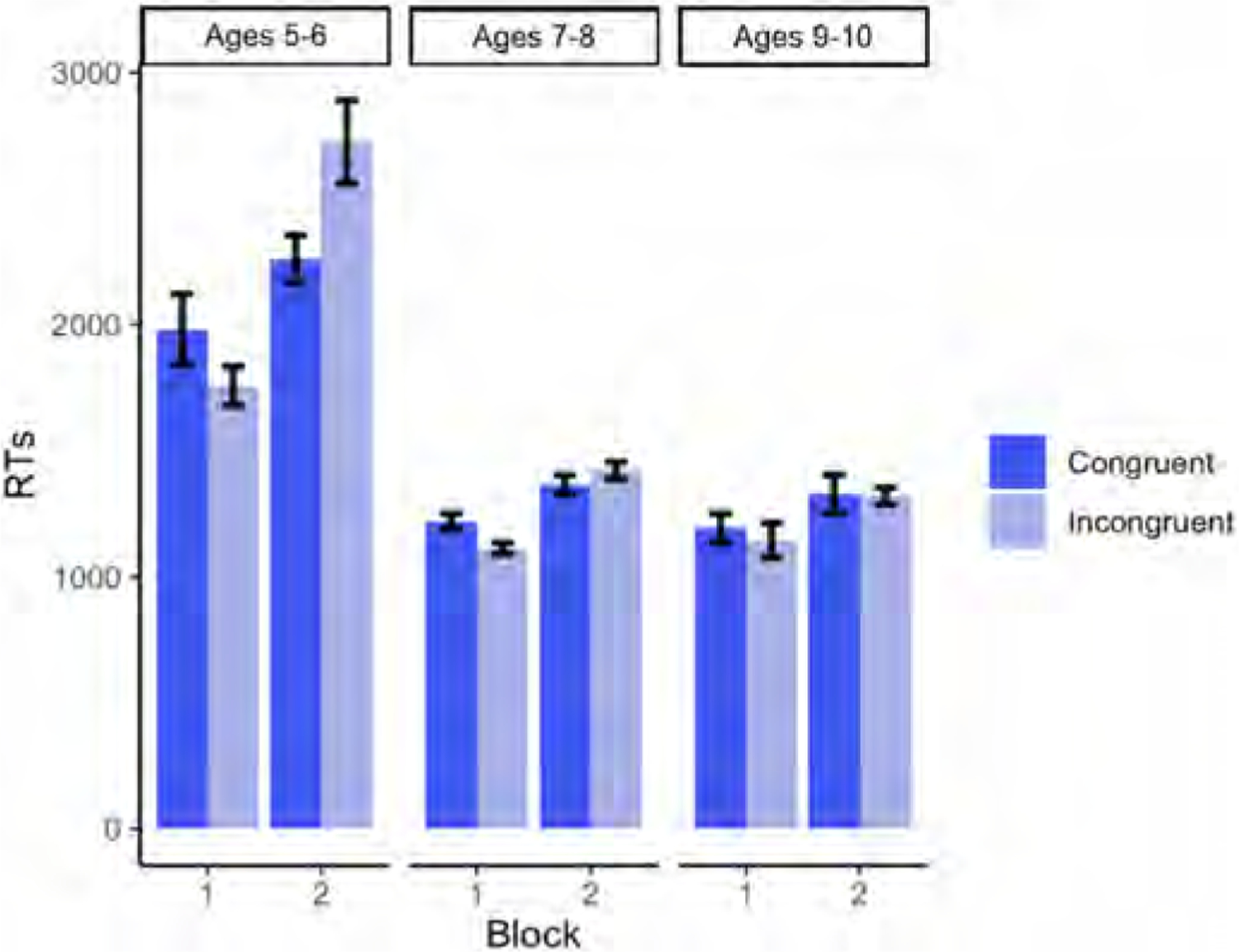
Average RTs (in milliseconds) on congruent and incongruent trials from Experiment 2, split by Block (Block 1 = self-perspective task; Block 2 = perspective-taking task) and Age.

Descriptive statistics for each age group’s RTs (in milliseconds) on congruent and incongruent trials split by block are shown in Fig. 5.

**Fig. 6.**
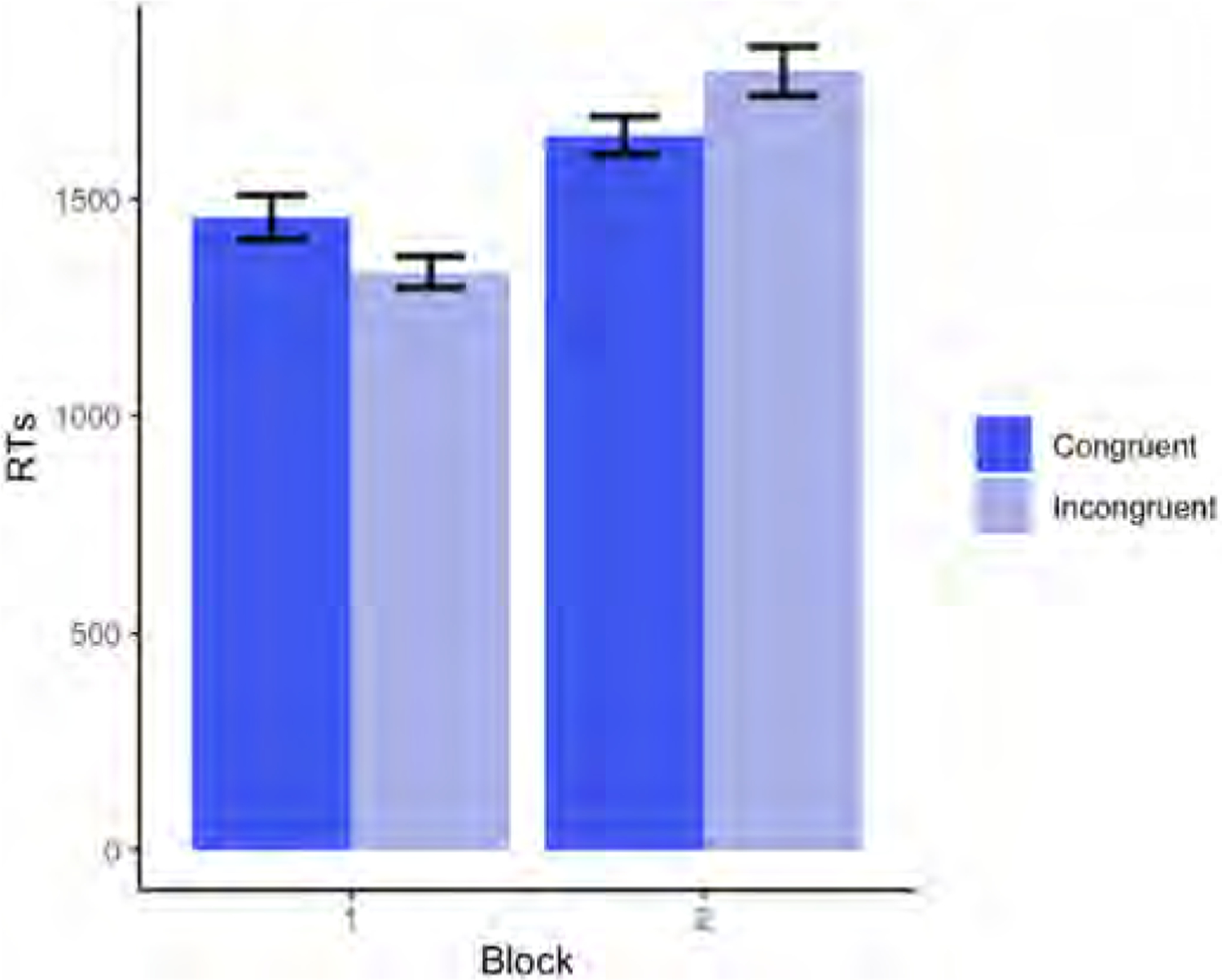
Block × Condition interaction for RTs (in milliseconds) in Experiment 2 (Block 1 = self-perspective task; Block 2 = perspective-taking task).

**Fig. 7.**
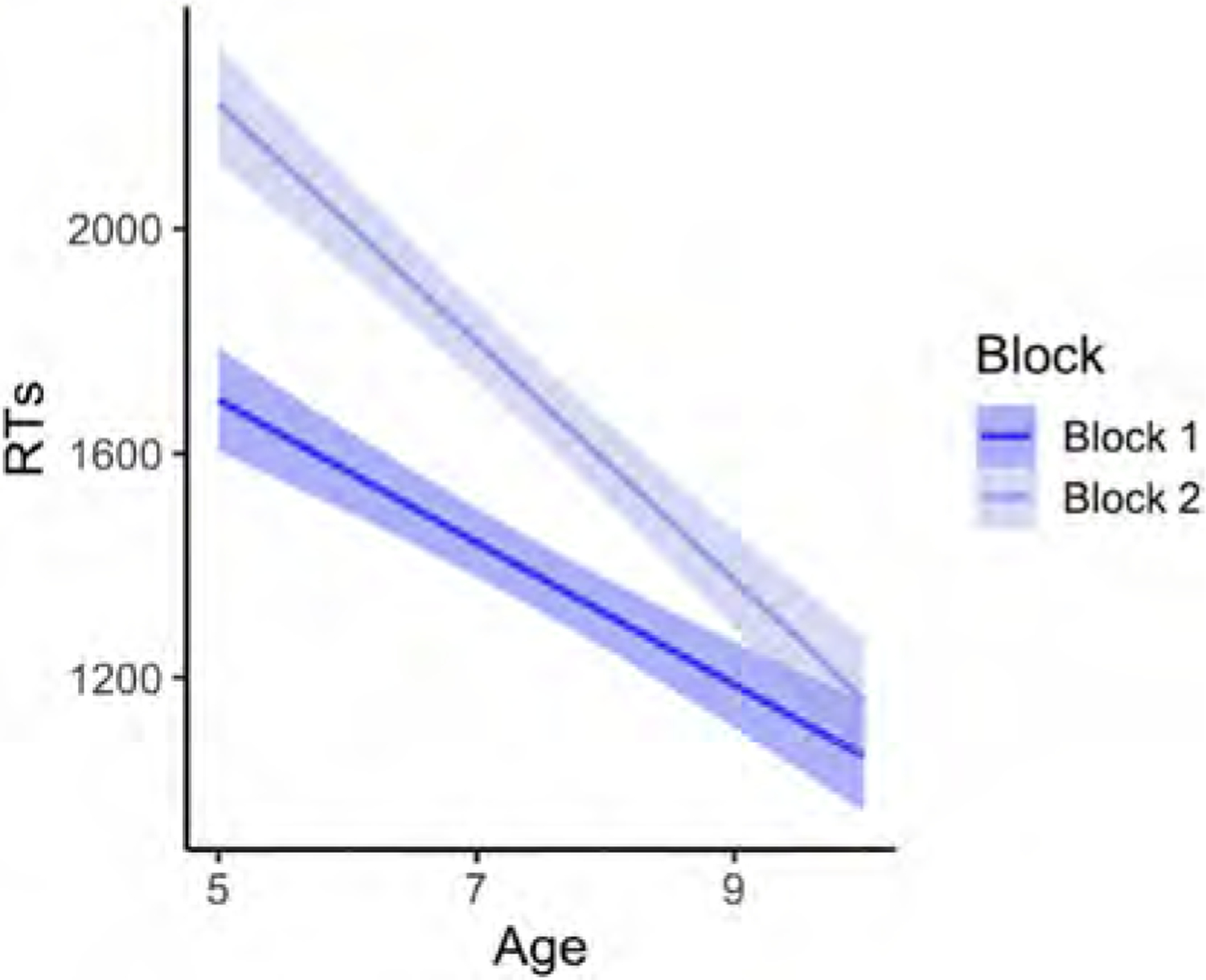
Block × Age interaction for RTs (in milliseconds) in Experiment 2 (Block 1 = self-perspective task; Block 2 = perspective-taking task). Regression lines reflect the best fit of data. The shaded bands around the regression lines represent a 95% confidence region for the regression fit.

**Fig. 8.**
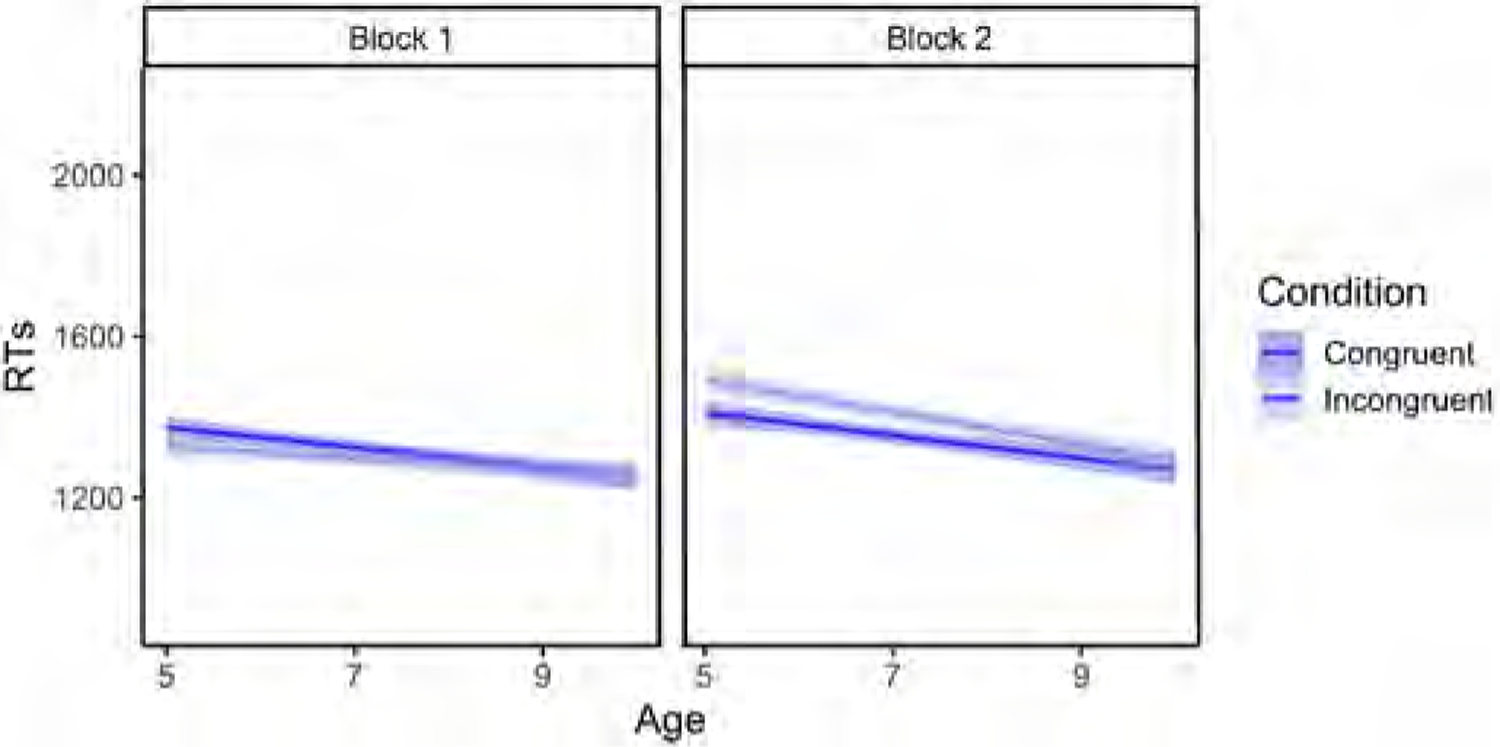
Block × Age × Condition interaction for RTs (in milliseconds) in Experiment 2 (Block 1 = self-perspective task; Block 2 = perspective-taking task). Regression lines reflect the best fit of data. The shaded bands around the regression lines represent a 95% confidence region for the regression fit.

Our results revealed a significant main effect of Age (p < .001), whereby RTs were faster as age increased (for the full model output, see Table 6). There was also a significant main effect of Block (p < .001), with faster RTs being recorded in Block 1. The Block × Condition interaction was also significant (p = .0261), revealing a difference in incongruent trials across blocks (p < .001), with slower RTs for incongruent trials being recorded in Block 2, but no difference across blocks for congruent trials (p = .189) (see Fig. 6). A significant Block × Age interaction (p = .0181) revealed that younger children’s RTs slowed down considerably in Block 2, whereas older children’s RTs were comparable across blocks (see Fig. 7). Finally, there was a significant Block x Condition × Age interaction (p = .0261), in which younger children’s RTs were slightly faster in incongruent than congruent trials in Block 1, but their RTs were faster for congruent trials in Block 2 (see Fig. 8).

## 7. Discussion

Experiment 2 did not reveal evidence of altercentric interference in any of the age groups: children from 5 to 10 years were able to perform the Dot task from their own perspective, without suffering interference from the agent’s view. This pattern of results may be explained by our task design, which did not require children to switch perspectives across trials (see also Ferguson et al., 2017; Surtees et al., 2016). It is therefore possible that, by not even mentioning it in the instructions, the agent’s visual perspective may not be salient enough to cause interference in the first half of the task (for comparable results with adults, see del Sette et al., 2021).

Regarding the two tasks administered in separate blocks, children were faster in Block 1 (self-perspective task) than in Block 2 (agent-perspective task). The significant effect of Block was further modulated by a significant interaction with Age, whereby younger children were slower than older children in the second half of the task (see Fig. 7). These two patterns of results confirm that there was a cost for adopting the agent’s perspective in the second block of trials, and that this cost was higher for younger children. However, the perspective-taking cost may have been accentuated by the block design, since children were asked to take the man’s visual perspective only after they had done the task from their own perspective.

Regarding a potential egocentric bias (i.e. lower accuracy rates and slower responses in incongruent trials than in congruent trials in the perspective-taking task in Block 2), the results confirmed that overall, children made more mistakes in the Incongruent condition in Block 2 than in Block 1, and were also slower in their responses (see Figs. 4 and 6). In addition, the greater difference between congruent and incongruent trials observed in Block 2 relative to Block 1 was further modulated by Age (see Fig. 8): younger children suffered more egocentric interference than older children when performing the task from the agent’s perspective.

Unlike Surtees and Apperly (2012), who did not observe a reduction of egocentric interference across ages, our simplified Dot task revealed two developmental patterns. First, by the age of 10, children were able to perform the task from the agent’s perspective as efficiently as they had performed the task from their own perspective in the first half of the task. Second, between the ages of 5 and 10, children went from suffering strong egocentric interference in the second half of the task, to performing comparably in congruent and incongruent trials in the two blocks. These results show that a sufficiently simplified version of the Dot task (simpler, at any rate, than the task design used by Surtees and Apperly (2012) with children of the same age) can reveal development of VPT in school children.

The question remains, however, as to whether adults would suffer altercentric interference in this version of the Dot task – perhaps because they have automatized VPT over years of social interaction, whereas primary school children may not have done so yet. Alternatively, the block design used in this task may reveal that adults do not necessarily suffer altercentric interference in the Dot task, challenging the automaticity view (see also Ferguson et al., 2017; del Sette et al., 2021). In addition, it would be interesting to see whether, in a very simple perspective-taking task, adults develop strategies during the course of the task to reduce both altercentric and egocentric biases.

## 8. Experiment 3

### 8.1. Methods

#### 8.1.1. Participants

Students at Jawaharlal Nehru University and University of Delhi (India) were contacted via email to take part in an online task for payment. The first 30 volunteers (aged 21–30) were recruited through this method before the link for the task was disabled.

#### 8.1.2. Materials, task design and procedure

The same materials, task design and procedure used in Experiments 1 and 2 were used again with adults in Experiment 3. The only difference was that the task was performed online individually, rather than on a Zoom call with an experimenter.

## 9. Results

### 9.1. Accuracy analysis

Using logistic mixed effects regression, we modelled the binary outcome variable of Response Accuracy (Correct = 1, Incorrect = 0) with Condition (Congruent, Incongruent), Block (Block 1 = Self perspective, Block 2 = Agent’s perspective) and Trial (1–80), and their interactions as predictor variables. Trial was entered as a scaled continuous predictor and deviation coding was used for Condition (Congruent = −0.5, Incongruent = 0.5) and Block (Block 1 = −0.5, Block 2 = 0.5). The model was fit with the maximal random effect structure for participants and items (Barr et al., 2013).

**Fig. 9.**
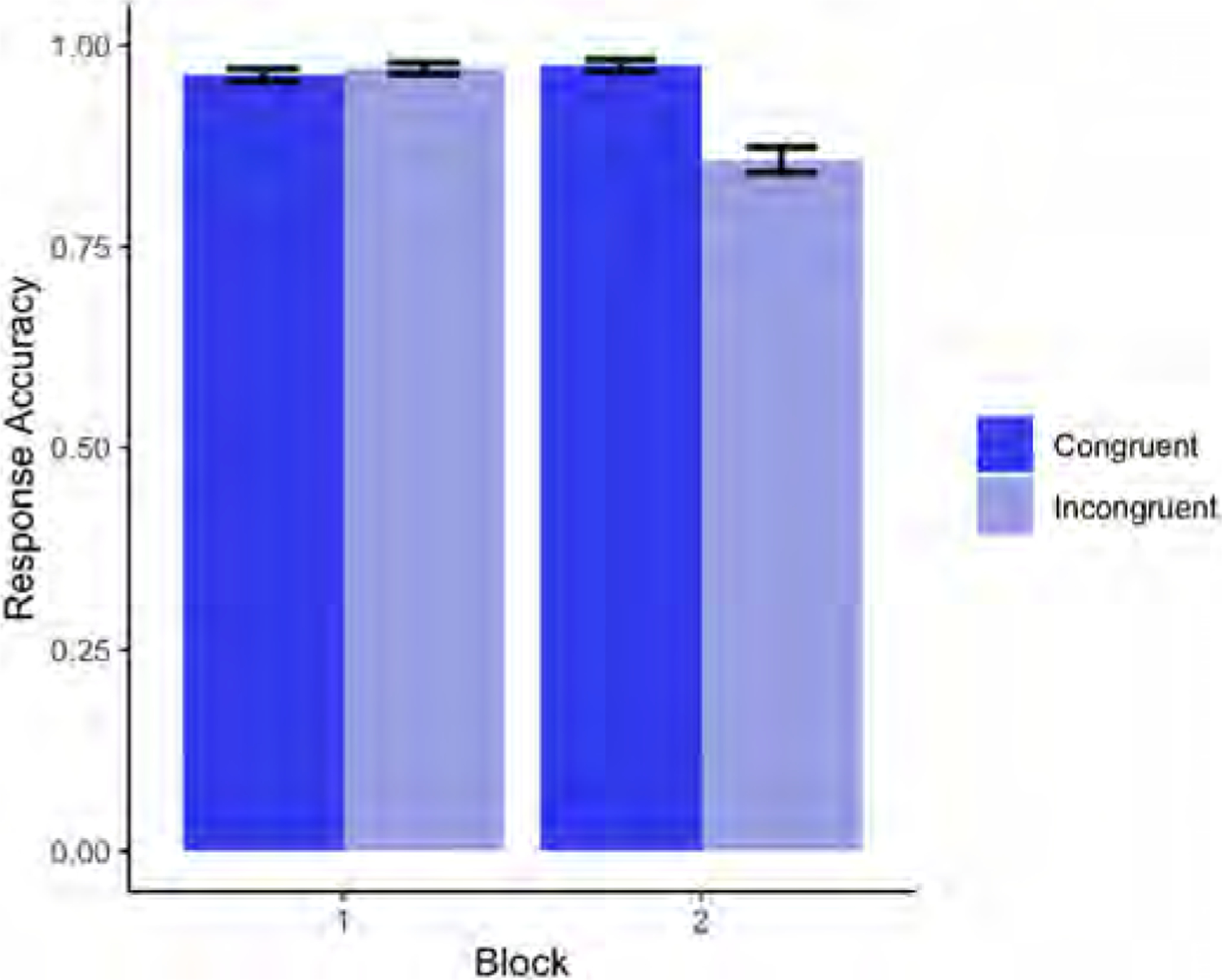
Mean proportion of accurate responses on congruent and incongruent trials from Experiment 3, split by Block (Block 1 = self-perspective task; Block 2 = perspective-taking task).

Descriptive statistics are shown in Fig. 9, revealing comparable accuracy rates in congruent and incongruent trials in Block 1 (self-perspective task), and a 12% decrease in incongruent trials, relative to congruent trials, in Block 2 (agent’s perspective task).

Model results did not reveal any significant main effects or interactions in the accuracy rates (for the full model output, see Table 7).

### 9.2. RT analysis

Using linear mixed effects regression, we modelled the outcome variable of RTs (for accurate responses) with Condition (Congruent, Incongruent), Block (Block 1 = Self perspective, Block 2 = Agent’s perspective), Trial (1–80), and their interactions as predictor variables. Trial was entered as a scaled continuous predictor and deviation coding was used for Condition (Congruent = −0.5, Incongruent = 0.5) and Block (Block 1 = −0.5, Block 2 = 0.5). The model was fit with the maximal random effect structure for participants and items (Barr et al.,2013).

**Fig. 10.**
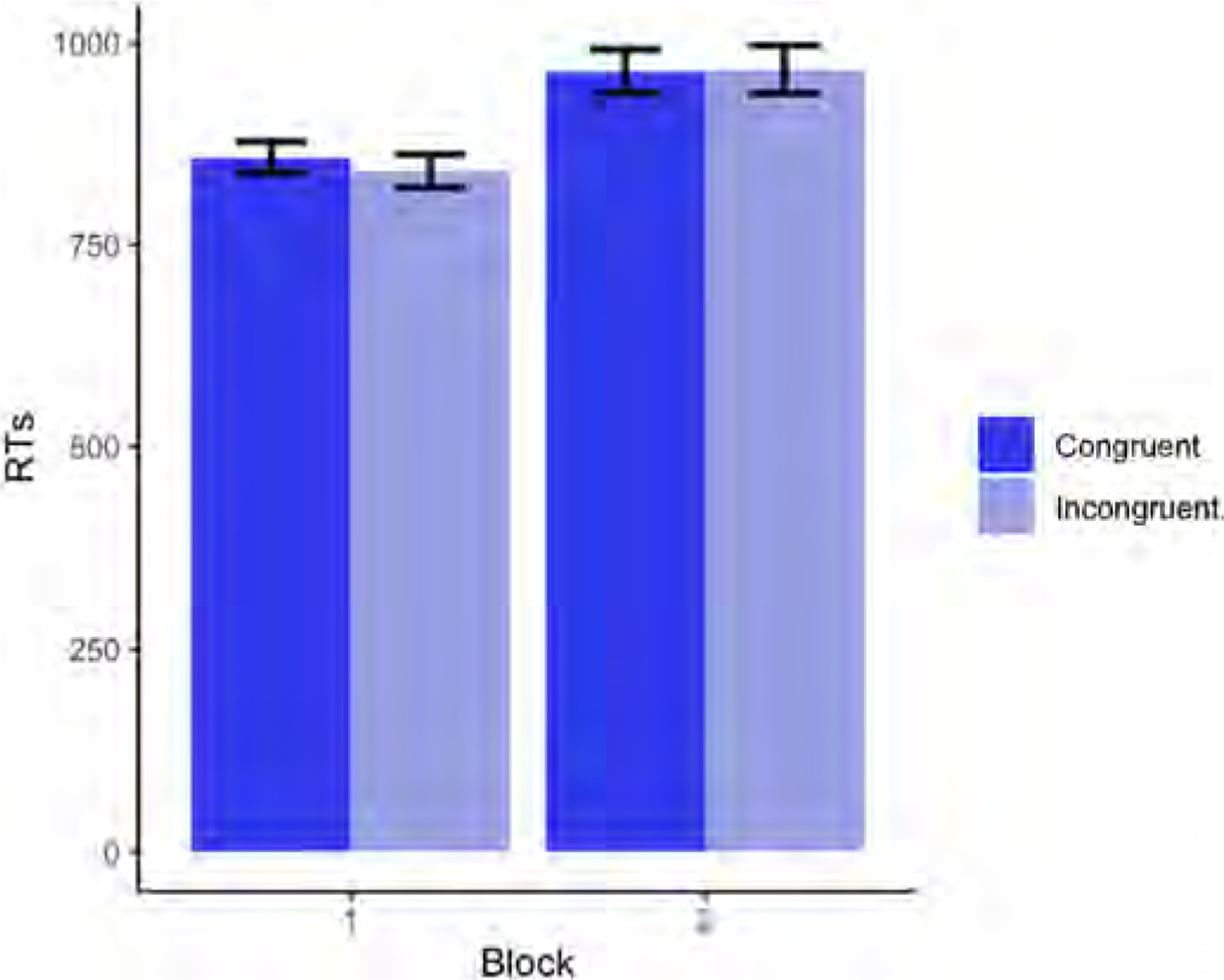
Average RTs (in milliseconds) on congruent and incongruent trials from Experiment 3, split by Block (Block 1 = self-perspective task; Block 2 = perspective-taking task).

Descriptive statistics for adults’ RTs (in milliseconds) on congruent and incongruent trials are plotted by block in Fig. 10.

Our results revealed a significant main effect of Block (p < .001), whereby RTs were faster in Block 1 than in Block 2 (for the full model output, see Table 8). There was also a significant main effect of Trial (p < .001), such that RTs became faster as the task progressed.

## 10. Discussion

Adults in this highly simplified version of the Dot task did not reveal either altercentric interference (Block 1) or egocentric interference (Block 2). However, RTs were slower in Block 2 than in Block 1, suggesting that performing the task from the agent’s perspective in the second half of the task was cognitively costly. The perspective-taking cost, however, might have been augmented by the change of instructions between the two blocks.

Overall, the results of Experiment 3 suggest that, while performing the Dot task from the agent’s perspective may come at a cost, when the task design is sufficiently simplified (with the two conditions administered in separate blocks and no working-memory load), adults do not show evidence of either altercentric or egocentric interference.

## 11. General discussion

Previous studies with the Dot task have revealed both altercentric and egocentric biases: participants suffered interference from the avatar’s view when performing the task from their own perspective, while their own view compromised their VPT when attempting the task from the avatar’s view. Here we used a highly simplified version of the Dot task that employed a block design (where participants first performed the task from their own perspective and adopted the agent’s perspective in the second half) with only two direct responses (1 or 2 dots) in order to adapt the task for newly sighted children. Unlike previous studies, we did not observe evidence of altercentric interference in any of the groups: Prakash children (Experiment 1), neurotypical children between the ages of 5 and 10 years (Experiment 2) and adults (Experiment 3), all performed the task from their own perspective without suffering interference from the agent’s view.

These findings suggest that previous evidence of altercentric interference may have resulted from employing a mixed-trial design, in which participants had to switch perspectives from one trial to the next. While altercentric interference has been observed with other designs (see Samson et al., 2010; Surtees et al., 2016), most Dot tasks required participants to switch perspectives across trials. Our results are in line with recent work by Ferguson et al. (2017) and del Sette et al. (2021), who observed that altercentric interference was reduced or even disappeared when participants could maintain their own perspective across trials.

While our participants did not suffer altercentric interference in the first block of trials, they revealed lower accuracy rates (Experiment 2) and longer RTs (Experiments 2 and 3) when taking the agent’s perspective in the second block of trials, suggesting that VPT is cognitively costly. It must be noted, however, that the perspective-taking cost may have been augmented by administering the perspective-taking task in the second block of trials.

Regarding egocentric interference, one of the Prakash girls in Experiment 1 revealed an egocentric bias in the perspective-taking task administered in the second block, with longer RTs in incongruent than congruent trials. This pattern of results is consistent with the developmental findings in Experiment 2: children ages 5–10 showed both lower accuracy rates and longer RTs in incongruent than congruent trials in the second half of the task. In addition, 5-year-olds showed greater egocentric interference than the older children, revealing longer RTs in the incongruent trials of the perspective-taking task.

These findings replicate two broad patterns of results observed in previous studies with the Dot task (see Qureshi et al., 2010; Nielsen et al., 2015; Ferguson et al., 2017, 2018; Furlanetto et al., 2016; Simpson and Todd, 2017): adopting someone else’s visual perspective is more taxing than relying on one’s own view, and the egocentric bias is normally stronger than the altercentric bias. Replicating these robust patterns of results confirms the validity of our simplified Dot task.

However, unlike Surtees and Apperly (2012), who report comparable levels of egocentricity in children 6–10 years and adults, here we observed two developmental patterns: first, the longer RTs observed in the second block (agent’s perspective) relative to the first one (self-perspective) were modulated by age, with older children performing more efficiently in the perspective-taking task than younger children. Second, the egocentric bias observed in a Prakash girl in Experiment 1 and the 5-year-olds in Experiment 2 decreased during development, with the 10-year-olds in Experiment 2 and the adults in Experiment 3 not showing evidence of an egocentric bias. Contrary to Surtees et al.’s conclusions, these results suggest that VPT does develop during childhood, with the characteristic egocentric bias of the early years diminishing over time (Piaget and Inhelder, 1956). These developmental patterns speak to the value of using a simplified design, rather than employing a cognitively costly one.

Finally, the comparable performance observed with Prakash girl C in Experiment 1 and the youngest neurotypical children in Experiment 2 offers support to the view of VPT as an emerging cognitive capacity that is dependent on social interaction and experience. Prakash girl C was 9 years old at the time of testing (see Table 1), with her biological age therefore matching the oldest children in Experiment 2. However, the egocentric interference observed in her performance was comparable to that of the 5-year-olds in our sample, which seems to suggest a protracted development of VPT in relation to peers her age (who did not suffer from an egocentric bias, similar to the adults in Experiment 3). Therefore, the results of our study support a developmental view of VPT, rather than a nativist one.

### 11.1. Issues of experimental design and ecological validity

The results of our study may be questioned on the grounds that the design of our Dot task was so simple that all participants were able to overcome their altercentric bias (which is normally weaker), while older participants were even able to overcome their egocentric bias (which is normally stronger). This interpretation of our findings rests on the assumption that the original design of the Dot task taps into an automatic process of VPT.

In earlier studies, participants were given a perspective plus a number of dots (e.g., ‘You see two’ or ‘He sees three’) and had to verify that information in the ensuing display. By contrast, our participants did not have to switch perspectives across trials and could respond with the actual number of dots that they themselves saw or the agent could see. We readily acknowledge that this is a much simpler experimental design that may have allowed our participants to overcome both their altercentric and egocentric biases, if they had any. However, assuming that was the case, our results raise an important question about the ecological validity of the standard Dot task: in what everyday circumstances do people suffer altercentric interference from others’ perspectives?

Given the results of previous studies, the automatic VPT observed in the Dot task requires participants to switch between their own perspective and the avatar’s across trials, while verifying in each trial a description stored in short-term memory. If perspective switching and memory load are ‘methodological requirements’ for observing automatic VPT in children and adults (with participants not revealing altercentric interference in simpler tasks), the implications of this automatic process for everyday life seem rather moot. Or to put it differently: the ecological validity of the standard Dot task as a test of automatic VPT in humans needs to be examined more closely.

In the simplified task used in this study, children and adults had to count or subitize the number of dots on a screen, either from their own perspective or from the man’s in the picture. Counting or subitizing is a much more common activity in everyday life than bearing in mind a certain description and verifying it against a visual display (which is the procedure of the standard Dot task). Therefore, even if we assume that the standard design taps into an automatic process (be that mentalizing or orientational), it is questionable whether and when people undergo such a process in their everyday lives.

On the other hand, our results confirm that counting or subitizing can be done without interference from another agent’s perspective (at least in a low range between 1 and 2 dots). Importantly, our results also confirm that counting or subitizing from another agent’s perspective is cognitively costly, with this perspective-taking cost decreasing with age in a simplified design.

Another difference between the simplified version of the Dot task used here and the original paradigm used in earlier studies was the depth of the visual scenes, with previous studies showing a full-body image of an agent, whereas the present study used a closer picture of an agent’s head and shoulders. While we acknowledge that the full-body orientation of an agent may be a more powerful orientational cue than only their head and shoulders, it must be noted that previous studies using full-body images of agents have also failed to observe altercentric interference when participants did not have to switch perspectives across trials (see Ferguson et al., 2017; del Sette et al., 2021). Therefore, the key methodological difference between studies observing altercentric interference with the Dot task and those failing to find such effects seems to be that the latter do not require perspective switching across trials, hence posing lower Executive Control demands on their participants.

In conclusion, if children and adults only suffer altercentric interference when performing perspective-taking tasks with high perspective-switching and working-memory demands, the automaticity of VPT in everyday life seems rather limited.

### 11.2. What do Theory of Mind tasks actually measure?

We are echoing here the title of Quesque and Rossetti’s (2020) review. In line with their conclusions, our results raise an important issue in Theory of Mind research; namely, the general tendency to over-interpret both children’s and adults’ performance as evidence of their social cognition skills, without a parallel assessment of the Executive Control demands of the task. This is a particularly problematic issue in Theory of Mind research because tasks in this field tend to contrast two different perspectives (e.g., the participant’s and the avatar’s in the Dot task), hence requiring not only social cognition skills, but also Executive Control (see Perner and Lang, 1999).

In the previous discussion, we assumed that the standard Dot task taps into an automatic process of VPT (albeit one with low ecological validity). Here we will explore the alternative possibility that the altercentric interference observed with the Dot task reflects an Executive Control failure, rather than automatic Theory of Mind. Under this view, the altercentric interference that some have interpreted as evidence of automatic VPT in humans may result from perspective-switching costs and short-term memory load. Compatible with this interpretation, our results suggest that when the avatar’s view is not made salient by intermixing perspective-taking trials in the task, it may not cause interference when participants perform the task from their own view (for similar results, see Ferguson et al., 2017; del Sette et al., 2021). Our results therefore disconfirm a rich interpretation of the results of the Dot task: the mere presence of another agent does not automatically trigger altercentric interference (cf. Surtees and Apperly, 2012; 2016).

In the case of the classic false-belief task, children have to predict where a protagonist will look for an object that has been transferred to a new location while the protagonist was away (Baron-Cohen et al., 1985; Wellman, 2014). Children under 4 years tend to fail this task, predicting that the mistaken protagonist will search in the new location. This pattern of results has been interpreted as evidence that preschoolers acquire a Theory of Mind around age 4 (Rakoczy, 2017). However, like the standard Dot task, false-belief tasks pose Executive Control demands that may compromise young children’s performance. For example, the standard test question ‘Where will Sally look for her doll?’ draws children’s attention to the current location of the doll, increasing the salience of the wrong response (for eye-tracking evidence with adults, see Rubio-Fernandez, 2013). When children are asked a more neutral question (e.g., ‘Where will Sally go now?’), 3 year olds are able to pass the false-belief task (Rubio-Fernandez and Geurts, 2013, 2016; although their performance may not reveal mental state attribution, see Rubio-Fernandez, 2019).

Another Theory of Mind task that has revealed an egocentric bias is the so-called *Director task* (Keysar et al., 2000, 2003). In this paradigm, participants need to take the speaker’s visual perspective in order to disambiguate their requests (e.g., ‘Move the small candle’), having to avoid interference from objects that are only visible to the participants (e.g., the smallest candle, which would be hidden from the speaker). Given the initial confusion that participants can experience in this task (which sometimes leads them to pick up the hidden object), some researchers have argued that people only use their Theory of Mind as a correction mechanism, often communicating from an egocentric perspective (Keysar et al., 2000; Apperly and Butterfill, 2009; cf. Nadig and Sedivy, 2002; Hanna et al., 2003).

However, as in the case of the Dot task and the classic false-belief task, the Director task also poses heavy demands on participants’ Executive Control. In particular, this paradigm rests on the highly artificial assumption that the speaker only knows about the objects they can see, and cannot refer to objects outside their visual field – a situation that very rarely applies in real life where we often talk about unseen objects. When the task is modified to avoid this unnatural assumption, people reveal sophisticated use of their Theory of Mind in the Director task (Rubio-Fernandez, 2017; Rubio-Fernandez and Jara-Ettinger, 2018; Jara-Ettinger and Rubio-Fernandez, 2021).

In summary, our results are compatible with an interpretation of the Dot task where the alleged evidence of involuntary VPT reveals, in fact, perspective-switching costs, rather than automatic mindreading processes triggered by the mere presence of an agent. This lean interpretation of the Dot task supports Quesque and Rossetti’s (2020) conclusion that the experimental methods employed in standard Theory of Mind tasks need to be more closely examined in order to better understand their results.

### 11.3. Implications for the domain-general view of the dot task

This study was designed to test the rich interpretation of the altercentric interference observed with the Dot task, as evidence of automatic VPT. The failure to replicate this effect with a highly simplified version of the original paradigm challenges the rich interpretation of earlier Theory of Mind studies. However, it must be noted that the present study did not aim to arbitrate between mindreading and domain-general interpretations of the altercentric interference observed in previous studies (e.g., Samson et al., 2010; Santiesteban et al., 2014). In that sense, our results challenge the general assumption that the Dot task taps into automatic cognitive processes (be those mentalistic or simply orientational), but does not elucidate what kind of mental representations or cognitive capacities underlie previous findings.

While not a direct test of the domain-general view of the altercentric interference observed with the Dot task, the results of our study have implications for that line of work. For example, in a recent study using the original paradigm by Samson et al. (2010), Vestner et al. (2022) observed comparable altercentric interference when participants were presented with a picture of a man in profile and a side picture of a desk fan. Desk fans are similar to arrows (which have been used as non-social cues in earlier studies with the Dot task; e.g., Santiesteban et al., 2014) in that they may serve as orientational cues without being agents; but unlike arrows, desk fans are not used by agents to signal direction (which makes them even ‘less social’ than arrows). In view of the comparable performance observed with humans and desk fans, Vestner and colleagues conclude that the altercentric interference observed in previous studies with the Dot task was the result of domain-general attention cueing, not automatic perspective taking.

Assuming that Vestner et al.’s (2022) conclusion is correct, our results call for future studies to investigate under what circumstances people suffer involuntary attention cueing. Since Vestner et al. used the original Dot-task paradigm, their experiments required participants to switch perspectives across trials and incur a working-memory load by bearing in mind a certain description that they had to verify against an upcoming image. Since our experiments did not reveal altercentric interference without these Executive Control demands (see also Ferguson et al., 2017; del Sette et al., 2021), the ecological validity of the Dot task remains under question – even as a non-mentalistic test of attention cueing. Future studies should therefore investigate the extent to which the congruency effects observed with this task are a result of perspective switching costs, rather than involuntary processes of attentional orienting.

## 12. Summary and conclusions

The three groups tested in our study with a highly simplified version of the Dot task – namely, newly sighted children, 5–10-year-old school children and adults, all showed evidence of perspective-taking costs in the second half of the task, but did not suffer altercentric interference when performing the task from their own perspective in the first half. In addition, one of the newly sighted girls and the youngest children in our neurotypical pool suffered from an egocentric bias that disappeared by age 9–10. Our results therefore suggest that VPT develops during childhood, probably supported by increasing experience with social interaction and the maturation of Executive Control.

For more than a decade, the results of the Dot task have been interpreted as evidence of automatic Theory of Mind by some researchers (e.g., Surtees and Apperly, 2012; 2016), while others understood it as a test of domain-general processes, such as attentional orientation (e.g., Santiesteban et al., 2014; Catmur et al., 2016; Vestner et al., 2022). Under an interpretation that assumes automatic processes of some kind (either mindreading or domain-general), our results call into question the ecological validity of the standard task because observing these involuntary responses seems to require additional switching costs and memory load (which are not characteristic of everyday situations of perspective taking or attentional orientation). Under a lean interpretation, the altercentric interference observed with the Dot task results from perspective-switching costs and memory load, rather than automatic Theory of Mind processes. In conclusion, the results of our study challenge the rich interpretation of the Dot task as tapping into involuntary processes of VPT, questioning the validity of the standard task as a direct test of Theory of Mind.

## Supplementary Material

Supplementary material

Appendix A. Supplementary data

Supplementary data to this article can be found online at https://doi.org/10.1016/j.neuropsychologia.2022.108256.

## Figures and Tables

**Table 1:** Demographic and clinical information for the Prakash girls in the study.

Participant	Age at surgery	Date of surgery	Type of cataract	Age at testing	PreOp Acuity (in logMAR)	PostOp Acuity (in logMAR)
A	8	Jan-19	Total anterior capsular	9	2.67	6mo: 2.16
B	8	Jan-18	Nuclear	10	1.87	12mo: 1.42
C	11	Jan-19	B/L	12	2.67	6mo: 2.67
D	11	Dec-12	B/L Membranous	18	FC@30 cm	Not available
E	12	Jan-19	B/L Membranous	13	1.53	6mo: 1.04

Note: FC = finger counting; B/L = bilateral.

**Table 2:** Model output for Participant A’s RTs in Experiment 1.

Fixed effect	Coefficient	SE	P-value
Block	−76.5150	136.0930	.7144
Condition	−224.4290	200.6330	.4785
Run	−488.4900	272.1860	.0025
Block × Condition	550.6390	88.6370	.2130
Block × Run	92.7460	401.2670	.5691
Condition × Run	−66.9670	161.0640	.7208
Block × Condition × Run	−17.5850	322.1280	.9568

Note: Significant main effects and interactions are shaded.

**Table 3:** Model output for Participant C’s RTs in Experiment 1.

Fixed effect	Coefficient	SE	P-value
Block	−201.0520	208.7040	.3363
Condition	508.4300	208.7040	.0155
Run	−487.3840	128.5070	.0210
Block × Condition	980.2960	417.4080	.0196
Block × Run	154.7560	226.1450	.5017
Condition × Run	−323.6130	257.0150	.2796

Note: Significant main effects and interactions are shaded.

**Table 4:** Model output for Participant E’s RTs in Experiment 1.

Fixed effect	Coefficient	SE	P-value
Block	−153.0600	129.8300	.2395
Condition	−19.7600	129.8300	.8791
Run	−152.8600	62.9800	.0159
Block × Condition	226.2500	259.6600	.3844
Block × Run	232.2200	125.9600	.0644
Condition × Run	4.5900	125.9600	.9710
Block × Condition × Run	181.2500	251.9100	.4725

Note: Significant main effects and interactions are shaded.

**Table 5:** Model output for response accuracy in Experiment 2.

Fixed effect	Coefficient	SE	P-value
Block	.0096	.3014	.9747
Condition	−.7418	.3787	.0501
Age	.4115	.1220	.0007
Block × Condition	−2.2037	.5860	.0002
Block × Age	−.0419	.1746	.8102
Condition × Age	−.0846	.1608	.5987
Block × Condition × Age	−.0652	.3184	.8376

Note: Significant main effects and interactions are shaded.

**Table 6:** Model output for RTs in Experiment 2.

Fixed effect	Coefficient	SE	P-value
Block	334.8890	73.1140	<.001
Condition	23.2270	75.1690	.7868
Age	−428.2430	79.691	<.001
Block × Condition	310.9750	62.972	.0261
Block × Age	−182.5120	118.693	.0181
Condition × Age	−53.4320	71.619	.5250
Block × Condition × Age	−296.0150	114.671	.0261

Note: Significant main effects and interactions are shaded.

**Table 7:** Model output for response accuracy in Experiment 3.

Fixed effect	Coefficient	SE	P-value
Block	−0.2334	1.6830	.8897
Condition	−0.7058	1.8107	.6967
Trial	2.2745	1.6816	.1762
Block × Condition	−3.6005	3.5621	.3121
Block × Trial	4.9256	3.3381	.1401
Condition × Trial	5.6429	3.2755	.0849
Block × Condition × Trial	9.0777	6.5890	.1683

**Table 8:** Model output for RTs in Experiment 3.

Fixed effect	Coefficient	SE	P-value
Block	876.02	96.59	<.001
Condition	56.44	47.35	.2519
Trial	−432.21	50.80	<.001
Block × Condition	109.55	84.70	.2003
Block × Trial	−78.81	59.08	.1922
Condition × Trial	−40.55	39.32	.3034
Block × Condition × Trial	−136.06	81.17	.0962

Note: Significant main effects and interactions are shaded.
