# Supplementary material for: Visual perspective taking is not automatic in a simplified Dot task: Evidence from newly sighted children, primary school children and adults
Source: Neuropsychologia. Author manuscript; Available in PMC 2025 Jul 14. (PMC12257553; doi:10.1016/j.neuropsychologia.2022.108256)
Supplement: Supplementary material [file NIHMS1809426-supplement-Supplementary_material.docx]

**Supplementary Materials**

*Visual perspective taking is not automatic in a simplified Dot task: Evidence from newly sighted children, primary school children and adults*

**Experiment 1**

**RT analyses**

Results revealed no significant main effects or interactions for Participant B (for the full model output, see Table S1).

*Table S1.* Model output for Participant B’s RTs in Experiment 1

| Fixed effect | Coefficient | SE | P-value |
| --- | --- | --- | --- |
| Block | 218.5480 | 140.0510 | .1733 |
| Condition | -43.4200 | 198.7780 | .8435 |
| Run | -142.9430 | 100.4740 | .2667 |
| Block x Condition | 12.0690 | 280.1020 | .9671 |
| Block x Run | -192.5690 | 141.1510 | .2259 |
| Condition x Run | 124.5690 | 200.948 | .5871 |
| Block x Condition x Run | 132.1140 | 282.302 | .6578 |

Results revealed no significant main effects or interactions for Participant D (for the full model output, see Table S2).

*Table S2.* Model output for Participant D’s RTs in Experiment 1

| Fixed effect | Coefficient | SE | P-value |
| --- | --- | --- | --- |
| Block | 106.2870 | 60.7960 | .0970 |
| Condition | -138.1200 | 63.1320 | .1362 |
| Run | -6.1840 | 29.9520 | .8366 |
| Block x Condition | 89.8170 | 121.5920 | .4694 |
| Block x Run | -46.3480 | 59.8900 | .4397 |
| Condition x Run | -17.0090 | 59.9030 | .7767 |
| Block x Condition x Run | 65.3070 | 119.7810 | .5860 |
